# Orthopedic manifestations and management of nail-patella syndrome: a narrative review

**DOI:** 10.3389/fped.2026.1744875

**Published:** 2026-02-04

**Authors:** Amandine Beaugé, Viola Sbampato, Oscar Vazquez, Giacomo De Marco, Christina Steiger, Romain Dayer, Dimitri Ceroni

**Affiliations:** 1 Faculty of Medicine, Imperial College London, London, United Kingdom; 2 Paediatric Orthopedics and Traumatology Unit, Geneva University Hospitals and University of Geneva, Geneva, Switzerland

**Keywords:** Fong disease, glaucoma, iliac horns, knee, nail patella syndrome, nephropathy, onycho-osteodysplasia

## Abstract

Nail-patella syndrome (NPS) is a rare autosomal dominant disorder characterized by skeletal and renal abnormalities. Diagnosis is primarily clinical, based on four main features: nail dysplasia, patellar and elbow abnormalities, and the presence of iliac horns. NPS affects approximately 1 in 50,000 individuals. Its presentation is highly heterogeneous, even within families, which may delay recognition. Pathogenic variants have been identified in the LMX1B gene located on chromosome 9q34. Although best known for its orthopedic manifestations, NPS may also involve other systems, notably the kidneys and eyes, leading to nephropathy or glaucoma that can progress to severe morbidity. Clinical features may be apparent from birth but will evolve over the life course, thus highlighting the need for long-term monitoring. Management requires a multidisciplinary approach, with orthopedics playing a central role. Surgical decisions, particularly for lower-limb pathology, is individualized and guided by symptom burden and functional impairment. This narrative review summarizes the current literature on NPS, including its epidemiology, etiopathogenesis, clinical manifestations, and natural history. It reviews diagnostic challenges based on clinical and radiologic features. Finally, it critically appraises reported surgical management strategies, with particular emphasis on knee pathology and associated complications, with aims to guide current clinicians.

## 1 Introduction

Nail-Patella Syndrome (NPS), also known as hereditary onycho-osteodysplasia (HOOD) or Fong disease, is a rare autosomal dominant disorder characterized by a classic clinical tetrad of nail dysplasia, patellar abnormalities, typically hypoplasia or aplasia, elbow abnormalities, and the presence of iliac horns (1–4). The syndrome is estimated to affect 1 in 50,000 individuals, but may be higher due to the underdiagnosis of this variable condition (1, 3, 5, 6).

Among the hallmark features, musculoskeletal involvement is the most frequent, with patellofemoral pathology being the earliest and most disabling manifestation due to the symptoms created by its abnormal osseous morphology (4, 7). Patellar defects range from hypoplasia to aplasia, frequently resulting in anterior knee pain, joint instability, dislocation, and an increased risk of early-onset osteoarthritis. As a result, orthopedic surgeons are often the first specialists to encounter patients with NPS, particularly during adolescence or early adulthood when joint symptoms become more disabling.

Despite the central role of patellar abnormalities in NPS, other skeletal manifestations include elbow abnormalities such as radial head subluxation, spinal involvement including mild scoliosis or lumbar lordosis, and pelvic anomalies such as “iliac horns” (1). Shoulder girdle dysplasia with glenohumeral arthritis has also been described (8).

Furthermore, NPS may involve ocular, renal, and even neurological domains (1, 9–12). Open ocular glaucoma is the main ophthalmologic complication, while nephropathy occurs in up to sixty per cent of patients, often presenting as asymptomatic proteinuria but can progress to renal failure (1, 10, 11, 13–17). Neuropsychiatric associations such as attention-deficit/hyperactivity disorder (ADHD) and major depressive disorder (MDD) have also been reported (1, 12, 14).

NPS poses a distinct clinical challenge due to its rarity and broad spectrum of clinical manifestations, often requiring highly individualized management. Existing orthopedic literature is predominantly composed of case reports and small case series, typically limited in its follow-up. While a wide range of surgical techniques has been reported in both children and adults, standardized treatment pathways have yet to be established. This lack of systematic synthesis has contributed to variability in clinical practice, which the present review aims to address.

Articles were identified through a PubMed search and limited to English-language publications reporting original research. Studies directly describing orthopedic surgical interventions in patients with NPS were included. Given the rarity of the condition and the limited available literature, case reports were also considered eligible.

## 2 Historical review

The earliest known familial description of NPS, dates back to the early 19th century, by Chatelain in 1820, when he documented a case with distinctive features not grouped previously (18–21). Its hereditary nature was later recognized in 1883 by Pye-Smith, and further substantiated in 1897 by Little, who cited a report by Sedgwick describing a family, in which 18 individuals across four generations exhibited similar physical abnormalities, including nail dysplasia and absent or hypoplastic patellae (3, 22, 23).

The clinical spectrum of the condition expanded in the early 20th century. In 1909, Wrede was the first to document additional elbow deformities, thereby introducing upper limb involvement into the clinical picture (24). Further detailed accounts followed, notably by Osterreicher in 1931 and Turner in 1933, both of whom emphasized the triad of nail, knee, and elbow abnormalities, as well as the dominant hereditary nature of the syndrome (25, 26). Kieser in 1939, and later Fong in 1946, described the presence of “iliac horns”, further expanding the phenotypic picture (27). These findings were confirmed by Mino and Thompson in 1948 and 1949, solidifying the addition of the fourth feature. This finalized the defining tetrad of the syndrome today, consisting of nail dysplasia, patellar anomalies, elbow dysplasia, and iliac horns (21, 28, 29).

Systemic features of NPS, particularly renal involvement, were recognized in the mid-20th century. Hawkins and Smith in 1950 first reported nephropathy in NPS patients, with further necropsy evidence presented by Darlington in 1967 (30). Ophthalmological complications were linked to NPS from the late 20^th^ century by linkage studies (31, 32). These observations eventually led to the inclusion of renal and ocular complications as secondary but clinically significant features of the syndrome (13, 33).

Insights into the genetics of NPS emerged in the 1950s. Aschner considered that probably four genes were involved, one for each facet of the tetrad, and they were closely linked (34). In 1955, Renwick and Lawler conducted extensive family studies, demonstrating that phenotypic variations were caused by modifying genes at the same locus as the main gene (35, 36). Their landmark research also demonstrated the non-sex-linked autosomal dominant inheritance by establishing its genetic linkage with the ABO blood group system in affected families (36).

The molecular basis of NPS was finally mapped in 1998, when Dreyer et al. identified mutations in the *LMX1B* gene on chromosome 9q34 as the underlying genetic cause (37). Subsequent research by Bongers and colleagues expanded the mutational landscape, identifying over 180 distinct variants and contributing significantly to our current understanding of the syndrome's clinical heterogeneity (38–40).

## 3 Epidemiology

NPS is a rare congenital disorder with an estimated incidence of approximately 1 in 50,000 live births (1, 3, 5, 6). It affects individuals of all racial and ethnic backgrounds and shows no sex predilection (36). *De novo* mutations occur in about 12% of the patients with NPS, and research demonstrated that 50% of offspring of an affected individual are at risk of inheriting the condition, demonstrating an autosomal inheritance (1).

Due to its variable clinical expression and incomplete penetrance, the true prevalence of NPS may be underestimated, as many individuals, particularly those with milder forms or subtle symptoms, remain undiagnosed or misdiagnosed (1, 3, 5, 6). Familial clustering is common, and a detailed family history often aids in diagnosis. Despite its lack of uniformity, the hallmark features of NPS provide a recognisable clinical pattern which can aid early identification (1–4).

Epidemiological data are limited due to the scarcity of large cohort studies, and most available information comes from case reports, small series, and genetic family studies. Population-based screening is currently not feasible, and awareness among clinicians remains essential for timely diagnosis.

## 4 Etiopathogenesis

The underlying genetic defect of NPS has been localized on the distal end of the long arm of chromosome 9, specifically, a mutation in the LIM Homeobox Transcription Factor 1 Beta gene (*LMX1B*) occurring in the region 9q34.1 (35, 37, 38). This gene encodes a transcription factor known as LIM-homeodomain *(LIM-HD*), which is necessary for embryonic development, particularly in regulating the development of the dorsal mesoderm.

The *LMX1B* protein has two LIM domains (LIM-A and LIM-B), which facilitate protein-protein interactions, and a homeodomain responsible for binding to DNA. This enables the regulation of downstream developmental genes expressed in the dorsal limb mesenchyme, podocytes, and anterior eye structures. Consequently, mutations in the *LMX1B* gene impair limb development, glomerular basement membrane integrity, and anterior segment formation of the eye, resulting in the characteristic NPS phenotype (41–44).

Since the NPS genetic locus mapping in the 1990s, more than a hundred pathogenic mutations in *LMX1B* have been described, including missense, nonsense, frameshift, and splice-site mutations (6, 37, 45–47). These mutations often cluster within functional protein domains, but no clear genotype–phenotype correlation has been established (45–47).

The variability in NPS phenotype is most likely the result of haploinsufficiency, where a single normal gene copy produces insufficient proteins for normal development. However, haploinsufficiency alone does not account for the wide range of symptoms seen in affected individuals within the same family. Possible explanations include modifier genes, epigenetic influences, and regulatory region mutations that affect when and where *LMX1B* is expressed. The concept of isoalleles, proposed in 1956, also suggests that alleles inherited from different parents might modify phenotypic expression (6).

Animal studies provide further insight into the importance of a well-functioning *LMX1B* gene for dorsoventral patterning and renal morphogenesis. NPS features in *LMX1B*-deficient mice include limbs with dorsal-to-ventral transformations. For example, loss of dorsal features such as nails and hair follicles, and the ectopic appearance of ventral structures, like toepads on the dorsal side of the limb (47). Even deeper structures, including dorsal tendons and muscle masses, are duplicated ventrally, revealing a mirror-image symmetry not observed in wild-type embryos. In addition to limb defects, these mice also displayed renal abnormalities, including thickened and irregular glomerular basement membranes, closely resembling the nephropathy seen in human NPS (47). The severity of the nephropathy is thought to be linked with specific *LMX1B* mutations, but uncertainty remains (1, 14, 46).

Nevertheless, not all NPS anomalies result directly from *LMX1B* dysfunction in bone and muscle tissues. Many skeletal and muscular features likely arise secondarily from impaired tendon or joint development. For example, muscle hypoplasia and weakness may result from defective anchoring or reduced mobilization due to altered joint architecture or connective tissue patterning (48). This is supported by both animal models and human muscle biopsy findings, which show that *LMX1B* is not expressed in myocytes but rather in joint-forming regions and connective tissue progenitors (47, 48). This suggests that *LMX1B* influences muscle development indirectly, through its role in shaping the musculoskeletal environment. In the studied NPS patient, only mild atrophy was seen in the deltoideus muscle without signs of primary myopathy, reinforcing the idea that muscle symptoms in NPS reflect secondary effects rather than intrinsic muscle pathology (48).

In conclusion, NPS results from impaired *LMX1B* activity, affecting the development of the limbs, kidneys, and eyes. Although haploinsufficiency is a key pathogenic mechanism, the marked clinical heterogeneity likely arises from a complex combination of genetic and regulatory factors. Further research into these modifiers will be essential to improve early diagnosis and understanding of the full phenotypic spectrum of this syndrome.

## 5 Natural history

Even within the same family, NPS can present with marked variability, with affected individuals showing heterogeneous phenotypes despite sharing the same genetic background (49). NPS can be diagnosed prenatally (50), and certain characteristic features, such as iliac horns, may be detectable as early as the first months of life (51). Skeletal and joint changes in NPS (50) can be detected on radiographs and have been documented in patients from the neonatal period onwards, with some cases diagnosed within the first days of life (52). Nevertheless, diagnosis during infancy is uncommon, partly because nail and patellar abnormalities become more apparent as the child grows. Patellar abnormalities may not be evident until later, as ossification typically begins between the ages of 4 and 6 and is not completed until adolescence. As a result, patellar dysplasia becomes more evident in adulthood (4, 53). However, many patients present earlier with functional issues affecting the feet, knees, elbows, and hips (4).

Additionally, the clinical course of NPS is highly variable; while some individuals remain almost asymptomatic, others develop significant complications, including early-onset osteoarthritis due to joint abnormalities (54–57). Further complications progress over time, such as nephropathy, which can worsen with age and, in some cases, lead to end-stage renal disease (1, 46). Although renal failure most frequently develops after the fourth decade of life, severe disease and even fatal outcomes have been reported in children and young adults (58). NPS renal abnormalities showed focal or diffuse irregular thickening and segmental effacement of foot processes in the podocytes. However, no correlation between the severity of the lesions and the severity of the manifestation is established (58).

During pregnancy, most females with NPS will progress to term without major complications (59). However, given the potential for renal involvement, careful monitoring of renal function is recommended. This is particularly important because early renal impairment may mimic pre-eclampsia, and distinguishing between the two is critical for appropriate management (1). Some studies have noted a possible association between NPS, pre-eclampsia, and adverse pregnancy outcomes, including in-utero fetal death, although most pregnancies in NPS proceed without incident.

## 6 Clinical presentation

Patients with NPS typically present in childhood, often to orthopaedic teams as the first point of contact, with complaints involving the feet, knees, elbows and hips (49). As they are frequently the first ones to encounter these patients, a comprehensive understanding of NPS is essential to ensure early and accurate diagnosis (49). Diagnosis is primarily clinical and is supported by the presence of the classical tetrad of nail dysplasia, patellar abnormalities, elbow dysplasia, and iliac horns (4, 46).

Knee involvement is highly prevalent, occurring in up to 80% of individuals with NPS (7, 46). These range from milder degrees of hypoplasia to complete agenesis and predispose patients to patellar instability (present in up to 90% of cases), increasing the risk of subluxation and dislocations (7, 35, 49). In some patients, intra-articular abnormality in the form of a sagittal fibrous or synovial septum of the anterior knee may be present. This structure partitions the patellofemoral joint and prevents the hypoplastic patella from engaging the trochlea, aggravating maltracking and instability (46). Genu valgum deformities are also observed, with knee symptoms such as pain and instability being the most common presenting complaints (49).

Pelvic findings are characterised by the presence of iliac horns and are pathognomonic of NPS, present in around 70%–80% of cases (46, 49). They are bilateral, often palpable bony projections that arise from the posterior aspect of the iliac crest at the gluteus medius attachment site (60–62), and are typically of no clinical consequence, rarely requiring intervention (49, 61, 63).

Upper limb involvement, though less common, remains well-documented (46). Patients can present with capitellar hypoplasia of the radial head, cubitus valgus deformities, elbow flexion contracture, and cubital webbing (49). In rare cases, clavicular horns, which are analogous to iliac horns, have also been reported (63). Finally, spinal abnormalities, including scoliosis and lumbar lordosis, have been described in patients with NPS (49).

All these musculoskeletal manifestations can predispose patients to joint subluxations and dislocations, sometimes bilateral, impacting their function and quality of life (49). Clinical assessment should include a full musculoskeletal review, with particular focus on patellar stability via dynamic patellar tracking, and screening for joint subluxations (49).

Although musculoskeletal features are predominant in NPS, this syndrome can affect other systems, requiring multidisciplinary teams to follow these patients holistically (1, 46).

Nail abnormalities are often the earliest and most recognisable feature. Manifestations include anonychia, koilonychia, longitudinal striations, triangular and absent lunula (46). Changes are mostly bilateral and are more severe in the thumbnails and milder on the ulnar side of the hand. Despite their characteristic appearance, nail deformities typically do not result in functional impairment (49).

Renal involvement occurs in thirty to fifty per cent of patients, ranging from isolated proteinuria and nephropathy to renal failure. In past studies, up to sixty per cent of patients exhibit some degree of nephropathy, with initial symptoms of asymptomatic proteinuria, but can progress from chronic to end-stage renal failure in fifteen to thirty per cent, respectively (1, 10, 11, 13–17, 35, 46).

Ocular manifestations are found in around half of the affected individuals, particularly as intraocular hypertension, glaucoma or normal tension glaucoma (1, 9, 14, 35, 45, 46).

Although neurological involvement is rare, a small subset of patients may present with complications such as sensorineural hearing loss or epilepsy (1, 9–12, 14). In addition to these findings, there is growing recognition of psychiatric manifestations, and several studies have reported higher rates of attention deficit hyperactivity disorder and major depressive disorder among individuals with NPS compared to the general population (1, 12, 14). While the precise mechanisms remain unclear, these neuropsychiatric features may be linked to the broader role of the *LMX1B* gene in neural development and dopaminergic pathways.

## 7 Radiological investigations

Although the diagnosis of NPS is primarily clinical, radiological imaging is fundamental to search for skeletal anomalies and guide treatment decisions, for example, during preoperative surgical planning, and can help monitor complications.

Plain radiographic imaging can help NPS diagnosis by detecting iliac horns on pelvic x-rays. These are bilateral, cone-shaped bony projections that form the posterior ilia and are visible in approximately seventy to eighty per cent of patients (3, 62). Knee radiographs, also known as the “NPS knee” in past literature, include a constellation of characteristics such as a shallow trochlear groove, a short and prominent anterior surface of the lateral femoral condyles, while the medial femoral condyle has a flattened anterior surface of the medial femoral condyle (7, 41). The characteristic patella aplasia can also be classified using x-rays. It is important to note that complete agenesis differs widely between studies, ranging from 4% to 52%. However, the majority will have some degree of hypoplasia (54). The patella's morphology can be grouped via the Wiberg classification, with the current hypothesis that the shape of the patella could determine the degree of patellar instability, with the example in Hunter's cap-shaped patella (54). x-rays can also aid in investigating arthritic changes (41).

Computer Tomography (CT) can be helpful to assess complex joint anatomy or alignment when standard radiographs are inconclusive (64). CT also assists in pre-surgical planning by clarifying bone morphology and alignment, particularly in the pelvis, knee and spine (55, 65). Magnetic Resonance Imaging (MRI) is also valuable when evaluating soft tissue structures, when detecting patellar cartilage wear, trochlear dysplasia, meniscal anomalies, sagittal fibrous or synovial septum, and sometimes synovitis in the various bones and joints affected (56). This is particularly relevant during the pre-operative period to determine areas of ossification centers in children, not demonstrated in x-rays, when avoided, are associated with better surgical outcomes (66, 67).

NPS patients might have a lower hip bone mineral density compared to the general population, with a normal to osteopenia T-score. Despite only mild numerical changes, pathological fractures were significantly more common (66).

Ultrasounds are used mostly to screen for complications but have contributed to prenatal diagnosis by visualizing the iliac horns and absent patella by ultrasound as early as 18–36 weeks of gestation. However, these were found in the context of a strong familial history and are not routinely assessed as part of routine screening (50, 67, 68).

Renal ultrasonography can detect and monitor nephropathy, which may remain asymptomatic for years. However, this method is not routinely used (67). Instead, other non-radiological investigations, such as urinalysis or blood tests, are preferred to monitor for renal and ocular complications.

## 8 Management

The surgical management of NPS is primarily guided by symptom severity and its impact on quality of life, often assessed using validated scoring systems such as the Knee Injury and Osteoarthritis Outcome Score (KOOS) or the Kujala anterior knee pain score (54). The current consensus in the literature recommends early surgical intervention in pediatric patients with patellar instability, with operative management reported in 23%–63% of cases (4). In contrast, procedures performed later in life are often less preventive in nature and more frequently represent salvage operations or corrective osteotomies undertaken in response to long-term (49, 66, 71). Overall postoperative outcomes are generally favorable, with a near-full range of motion achieved by one year in many patients (70, 72).

The decision regarding unilateral versus bilateral surgery appears to be symptom-driven, with some patients opting for the surgery contralaterally after a successful first procedure (66), while others remain asymptomatic on the contralateral side. Nevertheless, a subset of patients may remain asymptomatic and may never require surgical intervention (57, 71).

Surgical intervention is mostly undertaken for recurrent lateral patellar dislocation with or without associated pain (69, 70). These procedures are technically challenging due to the underlying skeletal abnormalities of NPS, including hypoplasia, dysplasia, abnormal alignment, and generalized ligamentous laxity, with patellar resurfacing often not feasible (4, 72, 74). Intraoperative navigation or robotic assistance has been used to facilitate surgical accuracy in complex cases (65, 70).

Reported surgical techniques encompass soft-tissue realignment procedures, medial patellofemoral ligament (MPFL) reconstruction, quadricepsplasties for extension contractures, capsular release for flexion deformities, and, in advanced cases of NPS associated symptoms, partial or total knee arthroplasty (4, 49, 54, 57, 65). The rationale guiding the choice of procedure is not consistently detailed in the literature but generally results from the presentation and anatomic abnormalities identified on imaging. MPFL reconstruction has demonstrated favorable outcomes, including preservation of the range of motion and improvement in patient-reported symptoms (70). The autograft tendon of choice can vary, although one study hypothesized free gracilis tendon, as a double band, could allow a stronger grip, particularly relevant in the context of a smaller patella (70). The Leeds-Keio ligament has also been reported to have positive outcomes (71, 73). Finally, the Krogius-Lecène procedure has demonstrated long-lasting results with good functional outcomes 26 years after follow-up (57). Although a medial parapatellar approach is most commonly used (41, 70–72), satisfactory outcomes have also been described following lateral meniscectomy in selected cases (73), with combined proximal and distal realignment encouraged (49).

Many reports describe an initial arthroscopic approach to assess joint integrity, identify loose bodies and resect the septum, before proceeding to open surgery to allow adequate patellar realignment (69, 72). When a sagittal fibrous or synovial septum of the anterior knee is present, its resection, either alone or in combination with realignment procedures, has shown to improve patellofemoral congruence, reduce instability, and enhance knee function, particularly in younger patients (1, 5, 54, 74, 75). Interestingly, reformation of the synovial band after prior resection has been observed, occasionally necessitating repeat excision during subsequent procedures (71).

Despite surgical stabilization, some patients will continue to experience mild pain or crepitus, even in the absence of dislocation or subluxation (73). However, no matter the choice of technique, a minority will require revision surgery due to complications such as tendon rupture, persistent pain, or recurrent patellar instability (4, 27, 41, 49, 67). In cases requiring total knee arthroplasty, fabrication of a custom patellar component has been necessary due to insufficient patellar thickness for reasonable resurfacing (4). This approach has been associated with complications, including patellar tendon rupture following postoperative falls. Other studies have also reported postoperative tendon injuries, including patellar tendon rupture following tibial tubercle anteriorization that required semitendinosus augmentation and cerclage wire reinforcement (4), as well as recurrent medial meniscal tears after arthroscopic septum resection performed without concomitant patellar realignment (72). Less favorable results have been reported in different techniques such as ipsilateral lateral release, tibial tuberosity transposition, or bilateral Judet quadricepsplasties. In the latter case, however, further surgery was not required, with symptoms improving through conservative management (4).

Age may also influence recovery trajectories. Younger age at the time of surgery appears to be associated with better outcomes (57, 66), particularly when intervention occurs before adolescence (4, 71). Several pediatric cases report excellent long-term outcomes, including absence of pain, return to sports and no surgical complications at up to 26 years of follow-up (57, 66). Older patients may experience prolonged rehabilitation, with more reports of falls resulting in tendon rupture within months of surgery or insufficiency fractures in some cases (72). This may be of impact as subsequent repairs and revision procedures have led to persistent residual pain (4). However, we should note that although no comparative age-stratified studies exist, these findings may be purely incidental. Nonetheless, progression of symptoms into adulthood has been reported in non-operated knees (57).

Beyond the knee, orthopaedic management may include correction of foot deformities such as clubfoot, pes cavus, calcaneovalgus, congenital vertical talus, or equinus, which may be managed by early posteromedial release to achieve a plantigrade foot (49). Upper limb surgery is less common but may involve soft tissue releases or radial head excision, occasionally bilaterally (49, 76). Iliac horns may exceptionally require intervention (49).

A major limitation across the literature is the relatively short duration of follow-up for many reports. This is important as often the follow-up period may not reveal symptom recurrence that can manifest several years post-surgery (4).

Finally, as NPS is a multisystem disorder, medical management is also important. Renal involvement may require blood pressure control with angiotensin-converting enzyme (ACE) inhibitors, with progression to end-stage renal disease necessitating dialysis or transplantation. Ophthalmological complications, including glaucoma and ocular hypertension, may similarly require lifelong specialist monitoring and treatment, which may require laser treatment (77, 78).

## 9 Conclusion

To our knowledge, this review is the first to collate and discuss the range of orthopaedic management options described for patients with NPS, with particular emphasis on knee pathology. Potential publication bias exists within the current case report literature, as most papers describe successful surgical interventions with marked improvement in knee function and symptoms. However, larger longitudinal studies have also reported cases with complications or operative failures. Additionally, many studies lack long-term follow-up. This limits our ability to draw strong conclusions about the durability of outcomes and, therefore, the best surgical treatment. Given the wide variability of NPS manifestations, it is unlikely that a single surgical strategy will be universally applicable. A balance must be struck between treating current symptoms and preventing long-term complications. Standardized assessment tools, such as the Kujala or KOOS scores, may help guide management and evaluate outcomes more consistently across studies. Consensus seems to favor early surgical intervention in symptomatic patients, with arthroscopic followed by open approach. Ultimately, multidisciplinary research with longer follow-up is needed to establish evidence-based guidelines and help with the choice of surgical intervention and best timing, while recognizing the need for individualized treatment tailored to each patient's phenotype and functional demands.

## References

[1] Sweeney E Fryer A Mountford R Green A McIntosh I. Nail patella syndrome: a review of the phenotype aided by developmental biology. J Med Genet. (2003) 40(3):153–62. 10.1136/jmg.40.3.153 12624132 PMC1735400

[2] Bongers EM van Kampen A van Bokhoven H Knoers NV. Human syndromes with congenital patellar anomalies and the underlying gene defects. Clin Genet. (2005) 68(4):302–19. 10.1111/j.1399-0004.2005.00508.x 16143015

[3] Bongers E Gubler M-C Knoers N. Nail-patella syndrome. Overview on clinical and molecular findings. Pediatr Nephrol. (2002) 17(9):703–12. 10.1007/s00467-002-0911-5 12215822

[4] Louboutin L Wascher D Neyret P. Management of patellar problems in skeletally mature patients with nail-patella syndrome. Knee Surg Sports Traumatol Arthrosc. (2017) 25(10):3012–6. 10.1007/s00167-016-4044-y 26872454

[5] Doughty K Richmond J. Arthroscopic findings in the knee in nail-patella syndrome: a case report. Arthroscopy. (2005) 20(1):e1–5. 10.1016/j.arthro.2004.10.013 15650657

[6] McIntosh I Clough MV Schäffer AA Puffenberger E Horton V Peters K Fine mapping of the nail-patella syndrome. Am J Hum Genet. (1997) 60(1):133. 8981956 PMC1712569

[7] Tigchelaar S Rooy J Hannink G Koeter S van Kampen A Bongers E. Radiological characteristics of the knee joint in nail patella syndrome. Bone Joint J. (2016) 98(4):483–9. 10.1302/0301-620X.98B4.37025 27037430

[8] Loomer RL. Shoulder girdle dysplasia associated with nail patella syndrome. A case report and literature review. Clin Orthop Relat Res. (1989) 238:112–6. 10.1097/00003086-198901000-00017 2642772

[9] Lavorante NP Lester M Bonzaro C Bagnis A Traverso CE Cutolo CA. Nail- patella syndrome and glaucoma: a case report and review of the literature. Case Rep Ophthalmol. (2022) 13(3):984–90. 10.1159/000527234 36605036 PMC9808124

[10] Burghardt T Kastner J Suleiman H Rivera-Milla E Stepanova N Lottaz C Lmx1b is essential for the maintenance of differentiated podocytes in adult kidneys. J Am Soc Nephrol. (2013) 24(11):1830–48. 10.1681/ASN.2012080788 23990680 PMC3810075

[11] Edwards N Rice S Hynes AM. A novel LMX1B mutation in a family with end- stage renal disease of “unknown cause”. Clin Kidney J. (2015) 8:113–9. 10.1093/ckj/sfu129 25713721 PMC4310431

[12] López-Arvizu C Sparrow EP Strube MJ Slavin C Caroline D. Increased symptoms of attention deficit hyperactivity disorder and Major depressive disorder symptoms in nail-patella syndrome: potential association with LMX1B loss-of- function. Am J Med Genet B. (2010) 0(1):59–66. 10.1002/ajmg.b.31138 PMC3677769 21184584

[13] Bennett W Musgrave J Campbell R Elliot D Cox R Brooks R The nephropathy of the nail-patella syndrome. Am J Med. (1973) 54(3):304–19. 10.116/0002-9343(73)90025-9 4569963

[14] Bongers E Huysmans F Levtchenko E Genotype-phenotype studies in nail patella syndrome show that LMX1B mutation location is involved in the risk of developing nephropathy. Eur J Hum Genet. (2005) 13:935–46. 10.1038/sj.ejhg.5201446 15928687

[15] Granata A Nori G Ravazzolo R Marini M Castellino S Sicurezza E Nail- patella syndrome and renal involvement. Description of three cases and literature review. Clin Nephrol. (2008) 69(5):377–82. 10.5414/cnp69377 18538102

[16] McIntosh I Dunston JA Liu L Hoover-Fong EJ Sweeney E. Nail patella syndrome revisited: 50 years after linkage. Ann Hum Genet. (2005) 69:349–63. 10.1111/j.1529-8817.2005.00191.x 15996164

[17] Meyrier A Rizzo R Gubler MC. Nail-patella syndrome, a review. Atlas of Genetic Diagnosis and Counseling. (1990) 2:133–40. 10.1007/978-1-4614-1037-9_172

[18] Roeckherath C. The nail patella syndrome. Fortschr Geb Rontgenstr. (1951) 75:700–4.14916993

[19] Jones C DIamond D Amirfeyz R Garan M. Nail-patella syndrome. Orthop Trauma. (2009) 23(5):362–4. 10.1016/j.mporth.2009.05.011

[20] Oon CS Koh CL. Onycho-osteodysplasia—the nail patella syndrome. A report of two cases. Singapore Med J. (1971) 12(5):300–3. 5134053

[21] Thompson EA Walker ET Weens HS. Iliac horns. An osseous manifestation of hereditary arthrodysplasia associated with dystrophy of the fingernails. Radiology. (1949) 53(88):88–92. 10.1148/53.1.88 18131936

[22] Little ME. Congenital absence or delayed development of the patella. Lancet. (1897) 150(3865):781–4. 10.1016/S0140-6736(00)64875-4

[23] West JA Louis TH. Radiographic findings in the nail-patella syndrome. Proc AMIA Annu Fall Symp. (2015) 28(3):334–6. 10.1080/08998280.2015.11929265 PMC4462213 26130880

[24] Berl W. Kongenitale erbliche luxation der patella nach aussen. Berliner Klinische Wochenschrift. (1909) 46:363.

[25] Osterreicher W. Family with anonychia, patellar abnormalities and dislocation of the radius: dominant characteristics over five generations. Zeitschrift für Menschliche Vererbungs. (1931) 15:465.

[26] Turner JW. A hereditary arthrodysplasia associated with hereditary dystrophy of the nails. JAMA. (1933) 100:882. 10.1001/jama.1933.02740120020008

[27] Chen H. Nail-Patella Syndrome. Atlas of Genetic Diagnosis and Counseling. New York, NY: Springer (2012). 10.1007/978-1-4614-1037-9_172

[28] Duncan J Souter W Hereditary onychoosteodysplasia. J Bone Joint Surg. (1963) 45B:252.

[29] Mino RA Mino VH Livingstone RG. Osseous dysplasia and dystrophy of the nails; review of the literature and report of a case. Am J Roentgenol. (1948) 60:633. 18895008

[30] Darlington D Hawkins C. Nail-patella syndrome with iliac horns and hereditary nephropathy: necropsy report and anatomical dissection. J Bone Joint Surg. (1967) 49(1):164–74. 6019383

[31] Lichter PR Richards JE Downs CA Stringham HM Boehnke M Farley FA. Cosegregation of open-angle glaucoma and the nail-patella syndrome. Am J Ophtal. (1997) 124(4):506–15. 10.1016/s0002-9394(14)70866-9 9323941

[32] Romero P Sanhueza F Lopez P Reyes L Herrera LC. 194 A>C (Q65P) mutation in the LMX1B gene in patients with nail-patella syndrome associated with glaucoma. Mol Vis. (2011) 17:1929–39. 21850167 PMC3154131

[33] Eisenberg K Potter D Bovill E. Osteo-onychodystrophy with nephropathy and renal osteodystrophy: a case report. J Bone Joint Surg. (1972) 54(6):1301–5. 10.2106/00004623-197254060-00025 4568821

[34] Aschner B. A typical hereditary syndrome. Dystrophy of the nails, congenital defect of the patella and congenital defect of the head of the radius. JAMA. (1934) 102:2017–20. 10.1001/jama.1934.02750240023009

[35] Gecheva NA Angelov V Kaneva R. Case report: multidisciplinary approach in diagnosis and treatment of fong disease. J Surg Case Rep. (2024) 2024(4):rjae207. 10.1093/jscr/rjae207 38605702 PMC11007641

[36] Renwick H Lawler SD. Genetical linkage between the ABO and nail-patella loci. Ann Hum Genet. (1955) 19(4):312–31. 10.1111/j.1469-1809.1955.tb01356.x 14388536

[37] Dreyer S Zhou G Baldini A Winterpacht A Zabel B Cole W Mutations in LMX1B cause abnormal skeletal patterning and renal dysplasia in nail patella syndrome. Nat Genet. (1998) 19(1):47–50. 10.1038/ng0598-47 9590287

[38] Harita Y Kitanaka S Isojima T Ashida A Hattori M. Spectrum of LMX1B mutations: from nail–patella syndrome to isolated nephropathy. Pediatr Nephrol. (2017) 32(10):1845–50. 10.1007/s00467-016-3462-x 27450397

[39] Miao C Xiang X. A novel LMX1B mutation: nail-patella syndrome manifesting with isolated nail disorders. Int J Dermatol. (2022) 61(12):477–78. 10.1111/ijd.16239 35643888

[40] Leung LT Ho SKL Yiu WC Cheng SS Lo IFM Luk HM. Novel LMX1B variants in nail-patella syndrome. Hong Kong Med J. (2025) 31(1):75–7. 10.12809/hkmj2411483 39957108

[41] Yang J Heckmann ND Craig JD. Unicompartmental knee arthroplasty in a patient with nail patella sydnrome; A case report. JBJS Case Connect. (2020) 10:3. 10.2106/JBJS.CC.20.00216 32910587

[42] Curtiss J Heilig J. Delimiting development. BioEssays. (1998) 20(1):58–69. 10.1002/(SICI)1521-1878(199801)20:1<58::AID-BIES9>3.0.CO;2-O 9504048

[43] German D Manaye K White C Woodward DJ McIntire DD Smith WK. Disease-specific patterns of locus coeruleus cell loss. Ann Neurol. (1992) 32(5):667–76. 10.1002/ana.410320510 1449247

[44] Sánchez-García I Rabbitts TH. The LIM domain: a new structural motif found in zinc-finger-like proteins. TIG. (1994) 10(9):315–20. 10.1016/0168-9525(94)90034-5 7974745

[45] Schleutermann DA Bias WB Murdoch JL McKusick VA. Linkage of the loci for the nail-patella syndrome and adenylate kinase. Am J Hum Genet. (1969) 21(6):606. 5365763 PMC1706472

[46] Ghoumid J Petit F Holder-Espinasse M Jourdain A Guerra J Dieux-Coeslier A Nail–atella syndrome: clinical and molecular data in 55 families raising the hypothesis of a genetic heterogeneity. Eur J Hum Genet. (2016) 24:44–50. 10.1038/ejhg.2015.77 25898926 PMC4795216

[47] Chen H Lun Y Ovchinnikov D Kokubo H Oberg K Pepicelli C Limb and kidney defects in Lmx1b mutant mice suggest an involvement of LMX1B in human nail patella syndrome. Nat Genet. (1998) 19(1):51–5. 10.1038/ng0598-51 9590288

[48] Paul L Schänzer A Depienne C. Lessons learned from a muscle study. Orphanet J Rare Dis. (2025) 20:384. 10.1186/s13023-025-03911-0 40721798 PMC12306095

[49] Guidera KJ Satterwhite Y Ogen JA Pugh L Ganey T. Nail patella syndrome: a review of 44 orthopaedic patients. J Pediatr Orthop. (1991) 11:737–42. 10.1097/01241398-199111000-00007 1960197

[50] Padmanabhan LD Yesodharan D Nampoothiri S. Prenatal diagnosis of nail patella syndrome: a case report. Indian J Radiol Imaging. (2017) 27(3):329–31. 10.4103/ijri.IJRI_438_16 29089684 PMC5644329

[51] Sartoris DJ Resnick D. The horn: a pathognomonic feature of paediatric bone dysplasias. Aust Paediatr J. (1987) 23(6):347–9. 10.1111/j.1440-1754.1987.tb00288.x 3435331

[52] Ogden JA. Radiology of postnatal skeletal development. Skeletal Radiol. (1984) 11:246–57. 10.1007/BF00351348 6729496

[53] Sasidharan R Gupta N Toteja N Yadav B. Diagnosis nail-patella syndrome: can it be so simple? BMJ Case Rep. (2021) 14:e241833. 10.1136/bcr-2021-241833 33692073 PMC7949362

[54] Tigchelaar S Lenting A Bonger E van Kampen A. Nail patella syndrome: knee symptoms and surgical outcomes. A questionnaire-based survey. OTSR. (2015) 101(8):959–62. 10.1016/j.otsr.2015.09.033 26596417

[55] Serrano AF Serrano PM. Total knee arthroplasty for osteoarthritis in a patient with nail patella syndrome – A case report. Int Med Case Rep J. (2021) 14:871–6. 10.2147/IMCRJ.S345262 35002335 PMC8721022

[56] Matsumoto K Matsumoto Y Nawachi S Asano Y Katayama Y Miyawaki Y The first presentation of a case of nail-patella syndrome newly diagnosed at the onset of rheumatoid arthritis: a case report. BMC Musculoskelet Disord. (2024) 25(12):139. 10.1186/s12891-024-07242-2 38355529 PMC10865650

[57] Beguiristáin JL de Rada PD Barriga A. Nail-patella syndrome: long term evolution. JPediatr Orthop B. (2003):13–6. 10.1097/01202412-200301000-00002 12488765

[58] Najafian B Smith K Lusco MA Alpers CFA. Nail-patella syndrome–associated nephropathy. AJKD atlas of renal pathology. (2017) 70(4):19–20. 10.1053/j.ajkd.2017.08.001 28941488

[59] Wood D Savihtri S Dinesh S. Nail-patella syndrome in pregnancy. Anaesthesia Cases. (2014) 2(1):11–3. 10.21466/ac.NSIP2.2013

[60] Fong EE. Iliac horns (symmetrical bilateral central posterior iliac processes). Radiology. (1946) 47(5):517. 10.1148/47.5.517 20274622

[61] Goshen E Schwartz A Zilka L Zwas S. Bilateral accessory iliac horns pathognomonic findings in nail-patella syndrome. Scintigraphic evidence on bone scan. Clin Nucl Med. (2000) 25(6):476–7. 10.1097/00003072-200006000-00020 10836702

[62] Sitaula S Sitaula S Khadka P Adhikari R. Radiological manifestations of nail- atella syndrome in a 56-year-old female: a case report. Radiol Case Rep. (2024) 19:4327–30. 10.1016/j.radcr.2024.06.058 39170778 PMC11338080

[63] Yarali HN Erden GA Karaarslan F Bilgiç SC Cumhur T. Clavicular horn: another bony projection in nail-patella syndrome. Pediatr Radiol. (1995) 25(7):549–50. 10.1007/BF02015792 8545188

[64] Reed D Nichols DM. Computed tomography of “iliac horns” in hereditary osteo-onychodysplasia (nail-patella syndrome). Pediatr Radiol. (1987) 17(2):168–9. 10.1007/BF02388103 3562114

[65] Vakharia RM Meneses ZA Ardeljan AD Roche MW. Robotic-Assisted lateral unicompartmental knee arthroplasty in a patient with nail-patella syndrome. Arthroplasty Today. (2021) 8:171–5. 10.1016/j.artd.2021.02.018 33889699 PMC8049876

[66] Konrads C Reppenhagen S Plumhoff P Rudert M Steinert A Barthel T. Nail- patella-syndrome in a young patient followed up over 10 years: of the sagittal trochlear septum for patellofemoral pathology. SICOT-J. (2016) 2:26. 10.1051/sicotj/2016017 27247258 PMC4887663

[67] Gong Y Yang C Liu Y Liu J Qi X. Treatment of patellar instability in a case of hereditary onycho-osteodysplasia (nail-patella syndrome) with medial patellofemoral ligament reconstruction: a case report. Exp Ther Med. (2016) 6:2361–4. 10.3892/etm.2016.3180 PMC4888000 27284321

[68] Towers AL Clay CA Sereika SM McIntosh I Greenspan SL. Skeletal integrity in patients with nail patella syndrome. J Clin Endocrinol Metab. (2005) 90(4):1961–5. 10.1210/jc.2004-0997 15623820

[69] Feingold M Itzchak Y Goodman RM. Ultrasound prenatal diagnosis of the nail-patellar syndrome. Prenat Diagn. (1998) 18:854–6. 10.1002/(SICI)1097-0223(199808)18:8<854::AID-PD343>3.0.CO;2-S 9742578

[70] Pinette MG Ukleja M Blackstone J. Early prenatal diagnosis of nail.patellar syndrome by ultrasonography. J Ultrasound Med. (1999) 18:387–9. 10.7863/jum.1999.18.5.387 10327020

[71] Lippacher S Mueller-Rossberg E Nelitz M. Correction of malformative patellar instability in patients with nail-patella syndrome: a case report and review of the literature. Orthop Traumatol Surg Res. (2013) 99(6):749–54. 10.1016/j.otsr.2013.03.031 24029584

[72] Courington R Kerr M Adigweme O. Total knee arthroplasty using computer- assisted navigation in a patient with nail-patella syndrome. JBJS Case Connect. (2023) 13(3):e22.00679. 10.2106/JBJS.CC.22.00754 37527396

[73] Ishibashi T Tomita T Tamaki M Fujito T Odaka S. Total knee arthroplasty without reduction of the patella for genu valgum with permanent dislocation of the patella: a case of nail patella syndrome. Arthroplasty Today. (2023) 43(4):101099. 10.1016/j.artd.2023.101099 PMC9922780 36793587

[74] Heckman DS McCoy AJ Spritzer CE Garrett WE. Intercondylar synovial septum in two patients with nail-patella syndrome. J Knee Surg. (2013) 26:S107–11. 10.1055/s-0032-1324809 23288746

[75] Curbo ME Park KJ Brown LD Incavo SJ. Total knee arthroplasty in a patient with nail-patella syndrome (NPS). Knee J. (2019) 26(1):273–8. 10.1016/j.knee.2018.11.014 30503662

[76] Juma AH. Nail-patella syndrome in Saudi Arabia with new features and surgical procedures: the first described study. Medscape Gen Med. (2004) 6(2):6. PMC1395803 15266233

[77] Nomura E Inoue M Kobayashi S. Bilateral recurrent patellar dislocation in a patient with isolated patella aplasia-hypoplasia. Arthroscopy. (2007) 23(10):1136.e1–4. 10.1016/j.arthro.2006.07.034 17916489

[78] Chen H. Nail-patella Syndrome. Atlas of Genetic Diagnosis and Counseling. 3 edn. (2017):2033–41. 10.1007/978-1-4939-2401-1_172

